# Compared to Intermittant Claudication Critical Limb Ischemia Is Associated with Elevated Levels of Cytokines

**DOI:** 10.1371/journal.pone.0162353

**Published:** 2016-09-09

**Authors:** Juho Jalkanen, Mikael Maksimow, Maija Hollmén, Sirpa Jalkanen, Harri Hakovirta

**Affiliations:** 1 Department of Vascular Surgery, Turku University Hospital and Turku University, Turku, Finland; 2 Medicity Research Laboratory, Department of Microbiology and Immunology, Turku University, Turku, Finland; Centro Cardiologico Monzino, ITALY

## Abstract

Critical limb ischemia (CLI) is the advanced stage of peripheral artery disease (PAD) and associated with an extremely poor clinical outcome. In order to understand the possible role of circulating cytokines and poor outcome associated with CLI we compared the circulating cytokine profile of patients with CLI against patients with intermittent claudication (IC). The levels of 48 circulating cytokines were examined in 226 consecutive patients with peripheral artery disease (PAD) admitted for elective, non-urgent, invasive treatment of IC or CLI. The PAD patient cohort was evenly distributed between subjects with IC (46.5%) and CLI (53.5%). As accustomed in PAD, CLI was associated with higher age, chronic kidney disease and diabetes when compared to IC (*P* < 0.01 for all). In multivariable linear regression modeling taking into account the baseline differences between IC and CLI groups CLI was independently associated with elevated levels of a large number of cytokines: IL-1β, IL-1ra, IL-2Rα, IL-4, IL-6, IL-10, IFN-γ, GM-CSF, G-CSF (*P* < 0.01 for all), and IL-2, IL-7, IL-12, IL-13, IL-17, bFGF, VEGF, SCGF-β (*P* < 0.05 for all). The current findings indicate that CLI is associated with a circulating cytokine profile, which resembles serious medical conditions such as severe pancreatitis, sepsis, or even cancer. Compared to IC, CLI is a systemic inflammatory condition, which may explain the extremely poor outcome associated with it.

## Introduction

Critical limb ischemia (CLI) is the advanced stage of peripheral artery disease (PAD) in which atherosclerotic occlusions of the lower limbs arteries cause insufficient flow to maintain viable peripheral tissue. Clinical manifestations include rest pain, non-healing ulcers, and gangrene. If revascularization is not performed the condition is associated with an extremely high risk of limb loss [1,2]. Even after successful revascularization this patient group is characterized with severe cardiovascular morbidity and increased mortality [3–5].

Despite major efforts PAD remains an unrecognized emerging health crisis [2,6,7]. Over 200 million people suffer from PAD worldwide, and its incidence and burden of death is constantly increasing along with aging, but also an increasing amount of young adults are affected [8,9]. Approximately 10% of people >65 years old and over 20% of people >80 years old suffer from the disease [6,10]. Both symptomatic and unrecognized asymptomatic PAD is associated with an increase in mortality [3,11–13], but most importantly cardiovascular mortality has been shown to be highest among patients with lower limb atherosclerosis when compared to atherosclerosis of other vascular beds [12,14], Among patients with lower limb atherosclerosis especially those suffering from CLI face a significantly worse outcome than those with a milder disease state, i.e. intermittent claudication (IC). Generally the 3-year survival rate of undivided PAD patient cohorts has been around 70%, but the majority of deaths are a result of CLI [3,13,15]. The mortality rate of patients with CLI can be up to four times higher when compared to patients with IC, and for some specific patient populations with CLI the 3-year survival rate can be as low as 33–35% [3,5].

To further understand the nature of CLI and possible inflammatory activity associated with it, we examined the circulating levels of 48 different cytokines, chemokines and growth factors in a prospectively gathered cohort of patients with diagnosed and symptomatic PAD and compared patients with IC and CLI against each other adjusting for age, co-morbidities, risk factors, and concomitant medication.

## Materials and Methods

### PAD patient cohort

The present study is a part of our on-going the PURE ASO Study approved by the local Ethical Committee of the Hospital District of South-West Finland. The patient cohort comprised of consecutive patients that were admitted to the Vascular Department in the Turku University Hospital for either endovascular or operative treatment of PAD, as described previously [16]. Shortly, during a one-year enrolment period (from February 2012 to March 2013) 227 suitable patients were screened. One patient declined and 226 patients gave their written informed consent. Pre-treatment serum was collected from each patient. A complete medical history including co-morbidities and cardiovascular risk factors were collected from the medical records and intense interviews. Upon admission limb ischemia was scored in all patients according to ankle-brachial index (ABI), symptoms and clinical manifestations according to the Rutherford classifications. Following, for the comparison circulating cytokine levels the patient cohort was divided into two groups: IC (Rutherford classes 1–3) and CLI (Rutherford classes 4–6).

### Healthy reference group for presumed normal cytokine values

The aim of this study was to compare the circulating cytokine profile of patients with IC against patients with CLI. However, to establish what can be presumed as a normal cytokine value and to enhance analytical rigidity, we performed the same analyses from 20 middle-aged (30–60 year old) non-smoking healthy volunteers of university personnel. All were screened and interviewed by a vascular surgeon for signs and symptoms of PAD and a duplex ultrasound of the lower limb arteries was performed to ensure that there were no detectable atherosclerotic lesions.

### Blood sampling and analysis of cytokines, chemokines and growth factors

Cytokine analyses using a multiplex assay kit (Bio-Rad Laboratories) has been previously described [17]. Briefly, all blood samples were drawn in the morning after at least 8 h of fasting. 9 mL of whole blood was collected in a serum sample tube and left to clot at room temperature while it was transported to the MediCity Research Laboratory, University of Turku. On arrival, it was centrifuged at 2000 *g* for 10 min, after which the serum was extracted and stored at -70°C until analyses. All analyses were performed at once with a magnetic bead suspension array kit of Bio-Plex Pro Human Cytokine 21- and 27-plex panels according to the manufacturers instructions except that the amount of beads, detection antibodies and streptavidin-phycoerythrin conjugate were used at half of their recommended concentration as described previously [17,18]. Results were analyzed using the Bio-Plex 200 System and Bio-Plex Manager 6.0 software (Bio-Rad Laboratories). The 21-plex panel included interleukin 1α (IL-1α), IL-2 receptor α (IL-2Rα), IL-3, IL-12p40, IL-16, IL-18, cutaneous T cell-attracting chemokine (CTACK), growth-regulated oncogene α (GROα), hepatocyte growth factor (HGF), interferon α2 (IFN-α2), leukemia inhibitory factor (LIF), monocyte chemotactic protein 3 (MCP-3), macrophage colony-stimulating factor (M-CSF), macrophage migration inhibitory factor (MIF), monokine induced by IFN-γ (MIG), β-nerve growth factor (β-NGF), stem cell factor (SCF), stem cell growth factor-β (SCGF-β), stromal cell-derived factor 1α (SDF-1α), tumor necrosis factor β (TNF-β), and TNF-related apoptosis inducing ligand (TRAIL). The 27-plex included IL-1β, IL-1 receptor antagonist, IL-2, IL-4, IL-5, IL-6, IL-7, IL-8, IL-9, IL-10, IL-12p70, IL-13, IL-15, IL-17, basic fibroblast growth factor (bFGF), eotaxin, granulocyte colony-stimulating factor (G-CSF), granulocyte-macrophage colony-stimulating factor (GM-CSF), IFN-γ, IFN-γ-induced protein 10, monocyte chemotactic protein 1 (MCP-1), macrophage inflammatory protein 1α (MIP-1α), MIP-1β, platelet-derived growth factor (PDGF), regulated on activation normal T cell expressed and secreted (RANTES), TNF-α, and vascular endothelial growth factor (VEGF). The persons performing the cytokine measurements were unaware of the baseline characteristics of the patients [17].

### Statistical analyses

Statistical analyses were performed in association with a professional statistical provider, 4Pharma Ltd. (Turku, Finland). All statistical analyses were performed using SAS JMP Pro (version 11.2). The prevalence of cardiovascular risk factors, medications, and other baseline characteristics are presented as percentages, and differences between IC and CLI groups were examined using the Chi-square test. Baseline characteristics of numeric values (age, blood pressure, cholesterol level etc.) were analyzed for normality using the Shapiro-Wilk test and differences between IC and CLI groups using the Student’s T-test. Original cytokine values are reported as median and inter-quartile range. The differences in marker values between IC and CLI groups were examined using the Wilcoxon rank-sum test and Kurskal-Wallis test when incorporating a healthy reference level. The association of the degree of ischemia with measured markers was tested using the Spearman’s correlation of ABI value and original cytokine values.

Multivariate modeling for each cytokine was individually performed using a linear regression model for logarithm-transformed cytokine values. At first, all baseline characteristics encompassing cardiovascular risk factors, used medication, the presence of critical ischemia (CLI) vs. intermittent claudication (IC), and the localization of significant atherosclerotic lesions were taken into account. Variables included in the model were selected using nonparametric methods. Variables with a test P value <0.15 were entered into the model. Dichotomous variables were tested using the Wilcoxon rank-sum test, and variables in more than two categories were tested using the Kruskal–Wallis test. The full model for each cytokine was constructed by fitting all the variables selected using the *P* value criterion. In addition to the full model, a reduced model was constructed by removing variables that showed little or no association with the explored cytokine in the full model. This model was defined by removing the least one significant variable from the model one by one until all remaining variables showed a *P* value <0.10. Thus, in the end the most decisive variables affecting each specific cytokine were left in the model. Model fit was inspected visually using studentized residuals showing a reasonable fit for all models.

## Results

### Baseline characteristics

The patient cohort consisted of 226 participants. All were of Caucasian origin. Overall mean age was 71.5 years (SD, ± 11.4; range, 46–93 years). Four patients were missing the data on Rutherford classification. They went through diagnostic angiography, but their claudication symptoms were not PAD related. Thus, the final study cohort comprised of 222 patients. The patient cohort was rather evenly divided by both sexes (54% male) and by patients suffering from IC (46.5%) or CLI (53.5%). The prevalence of cardiovascular risk factors together with concomitant medication amongst patient groups are presented in Table 1. Patients in the CLI group were slightly older (74y vs. 68y; *P* < 0.01) than patients in the IC group, and patients in the CLI group had more chronic kidney diseases (CKD), rheumatic diseases, and diabetes. Patients in the IC group, on the other hand, had more dyslipidemia and a history of smoking (Table 1). In concordance with the previous findings total cholesterol level was higher in patients with IC and creatinine in patients with CLI. The use of beta-blockers, furosemide and systemic cortisone was significantly higher in patients with CLI (Table 1).

**Table 1:** Baseline characteristics.

	Healthy	IC	CLI	*P* value*
N	20	101	121	NS
Male/female	55%/45%	55%/45%	58%/42%	NS
Age	40 (SD, 9.8)	68 (SD, 9.3)	74 (SD, 11.5)	<0.01**
History of smoking	1%	68%	50%	<0.01
Hypertension	1%	72%	75%	NS
Dyslipidemia	1%	40%	25%	<0.05
Diabetes	0%	27%	43%	<0.05
CKD	0%	11%	35%	<0.001
Rheumatic disease	0%	10%	20%	<0.05
**Clinical characteristics**				
ABI	NA	0.5 (SD, 0.2)	0.4 (SD, 0.3)	<0.01**
Toe pressure (mmHg)	NA	50 (SD, 24)	29 (SD, 20)	<0.001**
Systolic PB (mmHg)	121 (SD, 8)	148 (SD, 25)	150 (SD, 28)	NS**
Total cholesterol	5.25 (SD, 1.1)	4.6 (SD, 1.3)	4.0 (SD, 1.0)	<0.01**
Creatinine	84 (SD, 19)	83 (SD, 27)	106 (SD, 85)	<0.05**
**Medication**				
Statins	0%	62%	66%	NS
Aspirin	0%	55%	61%	NS
Beta-blockers	0%	49%	63%	<0.05
ACEi	0%	43%	46%	NS
CCBs	0%	32%	31%	NS
Furosemide	0%	15%	40%	<0.001
Warfarin	0%	17%	25%	NS
Nitroglycerin	0%	13%	26%	<0.05
Metformin	0%	22%	16%	NS
Cortisone	0%	9%	27%	<0.001
ARBs	0%	19%	17%	NS
Gliptins	0%	12%	12%	NS
DMARDs	0%	6%	12%	NS
Clopidogrel	0%	7%	11%	NS
Bisphosphonates	0%	5%	8%	NS

*Chi-square test for IC vs. CLI groups

** T-test for IC vs. CLI groups. Abbreviations: intermittent claudication (IC), critical limb ischemia (CLI), chronic kidney disease (CKD), anckle-brachial index (ABI), blood pressure (BP), angiotensin converting enzyme inhibitors (ACEi), calcium channel blockers (CCBs), angiotensin receptor blockers (ARBs), disease modifying anti-rheumatic drugs (DMARDs)

### Multiplex analyses in general

The measured values of MCP-3, IFN-α2, LIF, IL-1α, IL-3, IL-15, and TNF-β were mostly below a detectable limit, and did not provide a solid basis for further statistical analyses. The measured values for RANTES on the other hand were constantly over the desired measurable range, and it too was excluded from further statistical analyses. Otherwise, in general the cytokine values were very scattered and tailing to the higher end of the range amongst patients with PAD, and especially in the CLI group. In comparison, the cytokine levels of the healthy reference group were tightly and normally distributed. All detectable original cytokine values are presented in Table 2 as median and inter-quartile range together with an evaluation of differences between IC and CLI groups using the Wilcoxon rank sum test, and in addition the Kruskal-Wallis test when incorporating a presumed normal level using the healthy reference group. The majority of circulating cytokine levels was significantly higher in CLI patients when compared to patients with IC. For the majority of detected cytokines this phenomenon became stronger if in comparison a healthy reference level was added to the analyses, i.e. the majority of circulating cytokine levels increased steadily from the healthy reference group to claudicants and then again to patients suffering from critical ischemia. These cytokines included IL-1b, IL-1ra, IL-2, IL-2Rα, IL-4, IL-6, IL-10, IL-13, TNF-α, IFN-γ, MIP-1α, MIG, M-CSF, HGF, SCF, SDF-1α and β-NGF (Table 2).

**Table 2:** Measured non-transformed cytokine values as median and inter-quartile range (pg/mL).

	Healthy reference	IC	CLI	*P* value*	*P* value**
**IL-1b**	4.32 (4.00–4.54)	4.75 (4.19–5.30)	5.12 (4.3–6.15)	<0.05	<0.01
**IL-1ra**	88.8 (79.9–97.6)	106 (94–122)	116 (99–144)	<0.01	<0.001
**IL-2**	25.5 (22.9–28.2)	29.6 (25.1–34.3)	31.8 (27.1–41.0)	<0.05	<0.01
**IL-2Rα**	56.4 (32.2–79.7)	94.0 (77.3–126)	131 (95–182)	<0.001	<0.001
**IL-4**	6.35 (6.04–6.93)	6.73 (6.16–7.57)	6.99 (6.37–8.14)	<0.01	<0.01
**IL-5**	6.46 (1.78–2.90)	2.04 (1.60–2.88)	2.48 (2.04–2.99)	<0.05	NS
**IL-6**	10.8 (9.87–13.3)	14.1 (12.2–18.1)	17.5 (14.7–23.8)	<0.001	<0.001
**IL-7**	14.3 (12.4–16.4)	12.9 (10.7–17.0)	16.9 (12.9–21.3)	<0.001	<0.01
**IL-8**	30.8 (26.5–33.3)	46.4 (35.9–66.2)	50.4 (40.2–69.6)	NS	<0.001
**IL-9**	48.2 (34.5–64.0)	58.8 (47.2–68.0)	58.5 (48.4–76.2)	NS	<0.05
**IL-10**	5.70 (4.34–8.34)	8.77 (5.43–12.2)	10.3 (7.20–15.1)	<0.05	<0.001
**IL-12**	71.6 (51.1–91.4)	87.4 (58.3–127)	101 (71.3–141)	NS	<0.01
**IL-13**	7.88 (7.19–9.29)	8.10 (5.74–10.3)	9.22 (6.64–13.7)	<0.05	<0.05
**IL-16**	115 (80.1–158)	108 (75.6–131)	116 (83.3–170)	NS	NS
**IL-17**	97.8 (73.4–125)	98.5 (79.7–118)	110 (76.6–149)	NS	NS
**IL-18**	60.2 (30.4–73.5)	66.9 (52.0–88.0)	62.4 (45.0–92.1)	NS	NS
**Eotaxin**	121 (103–141)	145 (118–163)	147 (127–189)	NS	<0.01
**TNF-a**	75.3 (65.6–83.2)	80.0 (68.9–93.5)	86.4 (71.7–106)	<0.05	<0.01
**IFN-g**	203 (188–225)	236 (187–268)	268 (212–328)	<0.01	<0.001
**IP-10**	405 (320–480)	861 (583–1101)	935 (636–1386)	NS	<0.001
**CTACK**	1092 (962–1418)	1660 (1268–2045)	1733 (1319–2199)	NS	<0.001
**MCP-1**	25.5 (17.8–26.7)	26.9 (20.9–36.9)	27.9 (19.8–38.4)	NS	NS
**MIP-1a**	8.15 (7.26–9.42)	9.83 (7.94–11.7)	11.0 (8.92–14.0)	<0.05	<0.01
**MIP-1b**	180 (150–210)	261 (209–346)	266 (212–354)	NS	<0.001
**MIF**	66.5 (44.5–87.4)	108 (74.3–138)	110 (79.2–178)	NS	<0.001
**MIG**	523 (367–606)	1621 (972–2551)	2020 (1406–3082)	<0.05	<0.001
**M-CSF**	7.29 (5.46–15.1)	10.2 (5.65–14.1)	13.4 (6.40–20.2)	<0.01	<0.01
**GM-CSF**	37.2 (33.1–40.4)	37.9 (31.9–43.9)	38.7 (33.5–48.1)	NS	NS
**G-CSF**	208 (189–239)	183 (157–232)	203 (174–254)	NS	NS
**PDGF**	2358 (1978–2636)	2111 (1577–2521)	1884 (1395–2330)	NS	<0.05
**FGF**	140 (127–173)	145 (118–163)	147 (127–189)	NS	NS
**HGF**	551 (444–609)	726 (590–887)	802 (633–987)	<0.05	<0.001
**VEGF**	95.5 (76.8–135)	126 (68.9–176)	126 (84.3–186)	NS	NS
**SCF**	94.8 (77.1–122)	108 (83.9–137)	126 (95.8–172)	<0.01	<0.01
**SCGF-b**	19500 (14600–20700)	14500 (10600–20300)	15200 (11400–21400)	NS	NS
**SDF-1a**	60.8 (51.6–69.2)	53.0 (35.1–76.5)	70.4 (48.7–93.2)	<0.01	<0.01
**b-NGF**	1.48 (1.26–1.70)	1.89 (1.35–2.37)	2.13 (1.64–2.62)	<0.01	<0.001
**GROa**	64.1 (50.0–103)	60.4 (37.4–85.1)	71.1 (48.6–114.8)	<0.01	<0.05
**TRAIL**	117 (85.8–136)	123 (85.5–166)	105 (72.1–144)	NS	NS

*Wilcoxon rank sum test for IC vs. CLI groups

** Kruskal-Wallis test difference across all three groups

The levels of IL-8, IL-9, IL-12, Eotaxin, IP-10, CTACK, MIP-1β and MIF did not significantly differ between the IC and CLI groups, but showed a significant increasing trend toward CLI when the healthy reference group was added to the statistical analyses. The levels of IL-16, IL-17, IL-18, MCP-1, GM-CSF, G-CSF, bFGF, VEGF, SCGF-β and TRAIL did not differ between IC, CLI or the healthy reference group. Only PDGF showed a decreasing trend toward CLI when the three groups were compared with each other.

### The association of cytokine values with the degree of ischemia

Using Spearman’s correlation ABI values were tested against original cytokine values obtained from the patient cohort. Nearly all cytokines had a tendency to rise as ABI values declined, i.e. a negative correlation. Only PDGF, MIP-1b, IL-18, CTACK and TRAIL had a slightly positive, but insignificant correlation with ABI values (data not shown). For IL-4 (r = -0.16; P = 0.03), IL-6 (r = -0.17; P = 0.01), IL-8 (r = -0.15; P = 0.01), IL-12 (r = -0.18; P = 0.01), GM-CSF (r = -0.16; P = 0.01), TNF-α (r = -0.17; P = 0.01) and VEGF (r = -0.23; P = 0.001) the negative correlation with ABI values was statistically significant.

### Multivariable modeling reveals that CLI is independently associated with a variety of cytokines

Due to the skewed distribution of cytokine levels, prior to multivariable modeling, a logarithmic transformation of cytokine values were performed. Despite transformation two distinct outliers with persistent exponential values throughout the measured cytokines were identified and excluded from the final multivariable analyses. Both had rheumatoid arthritis, the other with accompanying vasculitis as mentioned earlier, and the other with an under treated rheumatic disease. For clarity, the backward eliminating multivariable linear regression model analyzed each cytokine individually using only the patient cohort in order to find out which factors had a decisive effect on the cytokine under observation. Factors included IC vs. CLI, cardiovascular co-morbidities and risk factors presented in Table 1, and concomitant medication.

**Fig 1:**
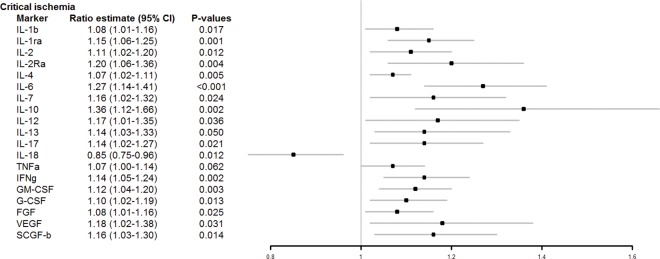
Cytokines associated with CLI when compared to IC. Forest plot illustrating the findings of multivariable modeling. The ratio value indicates that there is an independent 8% (95% CI, 1–16%) increase in the level of IL-1β associated with the presence of critical ischemia, an independent 15% (95% CI, 6–25%) increase in the level of IL-1ra in the presence of CLI, an independent 11% (95% CI, 2–20%) increase the level of IL-2 associated with the presence of critical ischemia etc.

According to the multivariable modeling, despite taking into account the baseline differences between the IC and CLI groups, CLI was independently and significantly associated with elevated levels of several cytokines (Fig 1). These include IL-1β (8% increase; 95% CI, 1–16%; *P* = 0.017), IL-1ra (15% increase; 95% CI, 6–25%; *P* = 0.001), IL-2 (11% increase; 95% CI, 2–20%; *P* = 0.012), IL-2Rα (20% increase; 95% CI, 6–36%; *P* = 0.004), IL-4 (7% increase; 95% CI, 2–11%; *P* = 0.005), IL-6 (27% increase; 95% CI, 14–41%; *P* < 0.001), IL-7 (16% increase; 95% CI, 2–32%; *P* = 0.024), IL-10 (36% increase; 95% CI, 12–66%; *P* = 0.002), IL-12 (17% increase; 95% CI, 1–35%; *P* = 0.036), IL-13 (14% increase; 95% CI, 2–27%; *P* = 0.05), IL-17 (14% increase; 95% CI, 2–27%; *P* = 0.021) and IFN-γ (14% increase; 95% CI, 5–24%; *P* = 0.002), which were already significantly elevated in univariate analyses. In addition, in multivariable modeling CLI was independently associated with elevated levels of GM-CSF (12% increase; 95% CI, 4–20%; *P* = 0.003), G-CSF (10% increase; 95% CI, 2–19%; *P* = 0.013), bFGF (8% increase; 95% CI, 1–16%; *P* = 0.031), VEGF (18% increase; 95% CI, 2–38%; *P* = 0.031), and SCGF-β (16% increase; 95% CI, 3–30%; *P* = 0.014), which did not show significantly different levels in univariate analyses of IC vs. CLI groups.

## Discussion

Atherosclerosis is contemporarily seen as an inflammatory disease [19,20] and there is a strong body of literature and reviews on the involvement of different cytokines, chemokines and growth factors in the inflammatory process of atherosclerosis [21–25]. The present study demonstrates that compared to IC, CLI is associated with markedly elevated levels of several cytokines, chemokines and growth factors. The wide range of circulating cytokines associated with CLI resembles more of a malignant inflammatory disease, e.g. severe pancreatitis [18], systemic inflammatory response syndrome [26,27], or even cancer [28,29], rather than just cytokines previously attached to the pathogenesis of atherosclerosis. Atherosclerosis and cancer have a lot of similarities in relation to risk factors and molecular pathology [30,31]. Atherosclerosis has even been considered a cancer of the artery wall [32,33]. In this respect, patients with CLI seem to have an aggressive and widely spread disease of the vasculature. However, together with the present observations and natural malignant course of CLI, it seems that the condition is not only dependent on inflammatory changes of the vascular wall, but also a state of systemic inflammation of the human body.

The burden of death of PAD has risen in both young and old populations and equivalent to major types of cancer such as prostate and breast cancer [9,34]. In comparison, only aggressive types of cancers, such as pancreatic cancer and lung cancer, have as poor short/mid-term survival rates as does CLI [34]. We wish to emphasize that patients in this study cohort do not carry malignant diseases. In addition, the current PAD patient cohort does not encompass the worst patients with CLI often coming in through the emergency room department, i.e. patients with infections, spread gangrene or severe tissue loss. These patients were excluded from the study, because the underlying acute conditions would significantly alter measured cytokine levels. Hence, the measured cytokine levels associated with CLI are not many times higher when compared to patients with IC, but they do present a significantly altered cytokine profile when comparing CLI and IC groups together.

Similar prior findings validate the current witnessed phenomenon. In a study of 14 different cytokines in 101 CLI patients compared to 37 healthy controls Teraa et al. reported that circulating levels of VEGF, SDF-1α, SCF, G-CSF, IL-6, IL-8 and IP-10 were significantly elevated in patients with CLI when compared to the healthy controls [35]. In another cohort of 293 PAD patients analysing only VEGF Stehr et al. showed that VEGF rises as the disease advances according to the Rutherford classification [36]. Findley et al. have also reported a similar phenomenon in a study of 23 patients with IC and CLI. They showed that plasma VEGF were elevated in patients with CLI when compared to patients with IC [37]. In this study a similar behaviour of serum VEGF was seen although no difference was seen in the univariate comparison of IC and CLI groups (Table 2). According to multivariable modelling VEGF was significantly higher in the CLI group when compared to the IC group (Fig 1), and VEGF increased along with disease severity as a measure of decreasing ABI values.

The virtue of this study is that it shows that within a substantial PAD patient cohort the cytokine profile associated with CLI is totally different even when compared to patients with IC. Out of 48 studied cytokines 28 differed significantly from healthy baseline values, 20 differed between IC vs. CLI groups in univariate analyses and a total 17 cytokines remained significantly altered in multivariable analyses between the IC and CLI groups. However, as seen from the comparison of original cytokine values there is overlapping in the range of cytokine levels, and no clear cut-offs between the groups exist. Thus, a single cytokine may not provide a useful diagnostic biomarker per se. The prognostic value of patterns of inflammatory cytokines in relation to patient outcome is currently an on-going study of ours.

## Conclusions

Even patients with a chronic stable state of CLI present a significantly altered cytokine profile when compared to patients with IC. In the future we need to understand is this ominous cytokine profile associated with CLI a result of the condition or the cause leading to it. In addition, we need to understand the course of these inflammatory pathologies in relation to successful or non-successful revascularization, wound healing, limb salvage and, on top of all, survival. The current findings help to explain the malignant cardiovascular behaviour and poor outcome associated with CLI, and open a new chapter in the fight against it. For a favourable outcome in this battle institutional level effort needs to be taken for the scientific and medical advancement in understanding and treating the condition.
